# Role of Recombinant DNA Technology to Improve Life

**DOI:** 10.1155/2016/2405954

**Published:** 2016-12-08

**Authors:** Suliman Khan, Muhammad Wajid Ullah, Rabeea Siddique, Ghulam Nabi, Sehrish Manan, Muhammad Yousaf, Hongwei Hou

**Affiliations:** ^1^The Key Laboratory of Aquatic Biodiversity and Conservation of Chinese Academy of Sciences, Institute of Hydrobiology, Chinese Academy of Sciences, Wuhan, Hubei 430072, China; ^2^Department of Biomedical Engineering, Huazhong University of Science and Technology, Wuhan 430074, China; ^3^Institute of Biotechnology and Genetic Engineering, The University of Agriculture, Peshawar 25000, Pakistan; ^4^National Key Laboratory of Crop Genetic Improvement, College of Plant Sciences and Technology, Huazhong Agricultural University, Wuhan 430070, China; ^5^Center for Human Genome Research, Cardio-X Institute, Huazhong University of Science and Technology, Wuhan 430074, China

## Abstract

In the past century, the recombinant DNA technology was just an imagination that desirable characteristics can be improved in the living bodies by controlling the expressions of target genes. However, in recent era, this field has demonstrated unique impacts in bringing advancement in human life. By virtue of this technology, crucial proteins required for health problems and dietary purposes can be produced safely, affordably, and sufficiently. This technology has multidisciplinary applications and potential to deal with important aspects of life, for instance, improving health, enhancing food resources, and resistance to divergent adverse environmental effects. Particularly in agriculture, the genetically modified plants have augmented resistance to harmful agents, enhanced product yield, and shown increased adaptability for better survival. Moreover, recombinant pharmaceuticals are now being used confidently and rapidly attaining commercial approvals. Techniques of recombinant DNA technology, gene therapy, and genetic modifications are also widely used for the purpose of bioremediation and treating serious diseases. Due to tremendous advancement and broad range of application in the field of recombinant DNA technology, this review article mainly focuses on its importance and the possible applications in daily life.

## 1. Introduction

Human life is greatly affected by three factors: deficiency of food, health problems, and environmental issues. Food and health are basic human requirements beside a clean and safe environment. With increasing world's population at a greater rate, human requirements for food are rapidly increasing. Humans require safe-food at reasonable price. Several human related health issues across the globe cause large number of deaths. Approximately 36 million people die each year from noncommunicable and communicable diseases, such as cardiovascular diseases, cancer, diabetes, AIDS/HIV, tuberculosis, malaria, and several others according to http://GlobalIssues.org/. Despite extensive efforts being made, the current world food production is much lower than human requirements, and health facilities are even below standard in the third-world countries. Rapid increase in industrialization has soared up the environmental pollution and industrial wastes are directly allowed to mix with water, which has affected aquatic marines and, indirectly, human-beings. Therefore, these issues urge to be addressed through modern technologies.

Unlike tradition approaches to overcome agriculture, health, and environmental issues through breeding, traditional medicines, and pollutants degradation through conventional techniques respectively, the genetic engineering utilizes modern tools and approaches, such as molecular cloning and transformation, which are less time consuming and yield more reliable products. For example, compared to conventional breeding that transfers a large number of both specific and nonspecific genes to the recipient, genetic engineering only transfers a small block of desired genes to the target through various approaches, such as biolistic and Agrobacterium-mediated transformation [1]. The alteration into plant genomes is brought either by homologous recombination dependent gene targeting or by nuclease-mediated site-specific genome modification. Recombinase mediated site-specific genome integration and oligonucleotide directed mutagenesis can also be used [2].

Recombinant DNA technology is playing a vital role in improving health conditions by developing new vaccines and pharmaceuticals. The treatment strategies are also improved by developing diagnostic kits, monitoring devices, and new therapeutic approaches. Synthesis of synthetic human insulin and erythropoietin by genetically modified bacteria [3] and production of new types of experimental mutant mice for research purposes are one of the leading examples of genetic engineering in health. Likewise, genetic engineering strategies have been employed to tackle the environmental issues such as converting wastes into biofuels and bioethanol [4–7], cleaning the oil spills, carbon, and other toxic wastes, and detecting arsenic and other contaminants in drinking water. The genetically modified microbes are also effectively used in biomining and bioremediation.

The advent of recombinant DNA technology revolutionized the development in biology and led to a series of dramatic changes. It offered new opportunities for innovations to produce a wide range of therapeutic products with immediate effect in the medical genetics and biomedicine by modifying microorganisms, animals, and plants to yield medically useful substances [8, 9]. Most biotechnology pharmaceuticals are recombinant in nature which plays a key role against human lethal diseases. The pharmaceutical products synthesized through recombinant DNA technology, completely changed the human life in such a way that the U.S. Food and Drug Administration (FDA) approved more recombinant drugs in 1997 than in the previous several years combined, which includes anemia, AIDS, cancers (Kaposi's sarcoma, leukemia, and colorectal, kidney, and ovarian cancers), hereditary disorders (cystic fibrosis, familial hypercholesterolemia, Gaucher's disease, hemophilia A, severe combined immunodeficiency disease, and Turnor's syndrome), diabetic foot ulcers, diphtheria, genital warts, hepatitis B, hepatitis C, human growth hormone deficiency, and multiple sclerosis. Considering the plants develop multigene transfer, site-specific integration and specifically regulated gene expression are crucial advanced approaches [10]. Transcriptional regulation of endogenous genes, their effectiveness in the new locations, and the precise control of transgene expression are major challenges in plant biotechnology which need further developments for them to be used successfully [11].

Human life is greatly threatened by various factors, like food limitations leading to malnutrition, different kinds of lethal diseases, environmental problems caused by the dramatic industrialization and urbanization and many others. Genetic engineering has replaced the conventional strategies and has the greater potential to overcome such challenges. The current review summarized the major challenges encountered by humans and addresses the role of recombinant DNA technology to overcome aforementioned issues. In line with this, we have detailed the limitations of genetic engineering and possible future directions for researchers to surmount such limitations through modification in the current genetic engineering strategies.

## 2. Recombinant DNA Technology

Recombinant DNA technology comprises altering genetic material outside an organism to obtain enhanced and desired characteristics in living organisms or as their products. This technology involves the insertion of DNA fragments from a variety of sources, having a desirable gene sequence* via* appropriate vector [12]. Manipulation in organism's genome is carried out either through the introduction of one or several new genes and regulatory elements or by decreasing or blocking the expression of endogenous genes through recombining genes and elements [13]. Enzymatic cleavage is applied to obtain different DNA fragments using restriction endo-nucleases for specific target sequence DNA sites followed by DNA ligase activity to join the fragments to fix the desired gene in vector. The vector is then introduced into a host organism, which is grown to produce multiple copies of the incorporated DNA fragment in culture, and finally clones containing a relevant DNA fragment are selected and harvested [11]. The first recombinant DNA (rDNA) molecules were generated in 1973 by Paul Berg, Herbert Boyer, Annie Chang, and Stanley Cohen of Stanford University and University of California San Francisco. In 1975, during “The Asilomar Conference” regulation and safe use of rDNA technology was discussed. Paradoxically to the view of scientists at the time of Asilomar, the recombinant DNA methods to foster agriculture and drug developments took longer than anticipated because of unexpected difficulties and barriers to achieve the satisfactory results. However, since the mid-1980s, the number of products like hormones, vaccines, therapeutic agents, and diagnostic tools has been developed continually to improve health [13].

**Figure 1:**
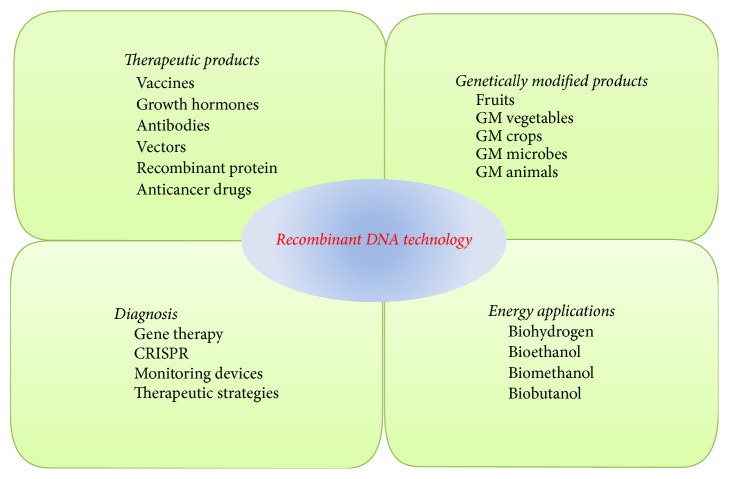
Illustration of various applications of recombinant DNA technology.

A quick approach is offered by recombinant DNA technology to scrutinize the genetic expression of the mutations that were introduced into eukaryote genes through cloned insulin genes insertion inside a simian virus fragment [3]. In a similar way, tumor growth was inhibited by adenoviral vector that encodes endostain human secretory form through antiangiogenic effects. Antiangiogenic effect can be enhanced by* dl*1520 through rescuing replication of Ad-Endo [14]. Targeted gene disruption has been used to produce antitumor derivatives in other hosts which were structurally similar for the production pathways [15]. Besides, longer acting therapeutic proteins have been developed through recombinant DNA technologies; for example, sequences containing additional glycosylation site are one of the most followed approaches. A new chimeric gene has been developed through this technique which contains the FSH *β*-subunit coding sequences and the C-terminal peptide of the hCG *β*-subunit coding sequences [16]. Researchers have also developed vectors and combined vectors for gene therapy and genetic modification approaches. Presently, viral vectors have received immense consideration in clinical settings, some of which have also been commercialized. In principle, viruses are modified to be safe for clinical purposes. They have several applications including treatment of severe diseases including cancer either through in vivo or gene therapy (ex vivo), vaccination, and protein transduction approaches [17]. The production of clinical grade viral vectors improvement has become possible due to advance manufacturing technologies [18]. At present, due to the severe adverse effects, retroviral vectors are losing their importance although the viral entities transfer genes quickly and correctly into a number of species. The simplest nonviral gene delivery system uses “naked” DNA, when injected directly into certain tissues, particularly muscles, produces significant levels of gene expression with least side effects [19]. More recently, a P1 vector has been designed to introduce the recombinant DNA into* E. coli* through electroporation procedures. This new cloning system is used for establishing 15,000 clone library initially averagely 130−150 kb pairs insert size. PAC cloning system is considered useful for complex genome analysis and in mapping [20]. The construction of low copy number vectors, for example, pWSK29, pWKS30, pWSK129, and pWKS130, was carried out using PCR and recombinant DNA technology. These vectors can also be used for generating unidirectional deletions with exonuclease, complementation analysis, DNA sequencing, and run-off transcription [21]. A broad range of applications of recombinant DNA technology has been summarized in Figure 1.

## 3. Current Research Progress

Recombinant DNA technology is a fast growing field and researchers around the globe are developing new approaches, devices, and engineered products for application in different sectors including agriculture, health, and environment. For example, Lispro (Humalog), in comparison with regular human insulin, is a well effective and fast acting recombinant insulin [3]. Similarly, Epoetin alfa is a novel and well-recognized recombinant protein that can be effectively used in curing of anemia [22]. Recombinant hGH was found with a great improvement in treating children lacking the ability to produce hGH in a required quantity. Clinical testing approval by the FDA in December 1997 for a recombinant version of the cytokine myeloid progenitor inhibitory factor-1 (MPIF-1) was an achievement to give recognition to this technology. With its help anticancer drug's side effects can be mitigated whereas it has the ability to mimic the division of immunologically important cells [23, 24]. The following section summarizes the most recent developments of recombinant DNA technology.

Clustered regularly interspaced short palindromic repeats (CRISPR), a more recent development of recombinant DNA technology, has brought out solutions to several problems in different species. This system can be used to target destruction of genes in human cells. Activation, suppression, addition, and deletion of genes in human's cells, mice, rats, zebrafish, bacteria, fruit flies, yeast, nematodes, and crops proved the technique a promising one. Mouse models can be managed for studying human diseases with CRISPR, where individual genes study becomes much faster and the genes interactions studies become easy by changing multiple genes in cells [25]. The CRISPR of* H. hispanica* genome is capable of getting adapted to the nonlytic viruses very efficiently. The associated Cas operon encodes the interfering Cas3 nucleases and other Cas proteins. The engineering of a strain is required with priming CRISPR for priming crRNAs production and new spacers acceptance. CRISPR-cas system has to integrate new spacers into its locus for adaptive immunity generation [26]. Recognition of foreign DNA/RNA and its cleavage is a controlled process in sequence-specific manner. Information related to the intruder's genetic material is stored by the host system with the help of photo-spacer incorporation into the CRISPR system [27]. Cas9t (gene editing tool) represents DNA endonucleases which use RNA molecules to recognize specific target [28]. Class 2 CRISPR-Cas system with single protein effectors can be employed for genome editing processes. Dead Cas9 is important for histone modifying enzyme's recruitment, transcriptional repression, localization of fluorescent protein labels, and transcriptional activation [29]. Targeting of genes involved in homozygous gene knockouts isolation process is carried out by CRISPR-induced mutations. In this way, essential genes can be analyzed which in turn can be used for “potential antifungal targets” exploration [30]. Natural CRISPR-cas immunity exploitation has been used for generation of strains which are resistant to different types of disruptive viruses [31].

CRISPR-Cas, the only adaptive immune system in prokaryotes, contains genomic locus known as CRISPR having short repetitive elements and spacers (unique sequences). CRISPR array is preceded by AT-rich leader sequence and flanked by cas genes which encode Cas proteins [32, 33]. In* Escherichia coli* cas1 and cas2 catalases promote new spacers through complex formation. Photo-spacer adjacent motif (PAM) is required for interference and acquisition because the target sequence selection is not random. The memorization of the invader's sequence starts after CRISPR array transcription into long precursor crRNA. During the final stages of immunity process, target is degraded through interference with invaded nucleic acids. Specific recognition prevents the system from self-targeting [32, 34]. In different species of* Sulfolobus*, the CRISPR loci contain multiple spacers whose sequence matches conjugative plasmids significantly while in some cases the conjugative plasmids also contain small CRISPR loci. Spacer acquisition is affected by active viral DNA replication in* Sulfolobus* species whereas the DNA breaks formation at replication forks causes the process to be stimulated [35]. According to the above information, CRISPR-Cas system has obtained a unique position in advanced biological systems because of its tremendous role in the stability and enhancement of immunity.

Zinc-finger nucleases (ZFNs) and transcription activator-like effector nucleases (TALENs) are chimeric nucleases composed of programmable, sequence-specific DNA-binding modules linked to a nonspecific DNA cleavage domain. Therapeutic potential of ZFNs and TALENs is more specified and targeted [25, 36, 37]. Similarly, recombinant protein fibroblast growth factor (FGF-1) has been developed which functions in inducing the formation of new blood vessels in myocardium. Its injection (biologic bypass) into a human myocardium cause an increased blood supply to the heart. Apligraf, an FDA approved product, which serves as a recombinant skin replacer, specified for the leg ulcer's treatment and DermaGraft, is effective in the treatment of diabetic ulcers [38–40]. After successful production of insulin from* E. coli* through recombinant DNA technology, currently several animals, notably cattle and pigs, have been selected as insulin producing source, which however, triggered immune responses. The recombinant human insulin is identical to human porcine insulin and comparatively infrequently elicits immunogenic responses. Furthermore, it is more affordable and can satisfy medical needs more readily. Human growth hormone was the first protein expressed in tobacco plants [41, 42]. Besides insulin, several new drugs related to recombinant DNA technology have undergone developmental improvements and a number of protein production systems have been developed. Several engineered microbial strains have been developed to carry out the formulation of drugs [41, 43, 44]. Molecular medicine formation that is specifically based on proteins faces serious issues including methods and biology of the cells which function to produce medically important compounds through recombinant DNA techniques. To overcome these obstacles, there is intense need to improve quality and quantity of medicines based on a molecular phenomenon. Cell factories are considered important in recombinant DNA technologies, but these needed to be explored with more details and in depth as the conventional factories are not fulfilling the needs [42]. Similarly, the endothelial growth factor and Notch signaling were used to engineer oncolytic adenovirus which acts as a breast cancer selective agent for the antagonist's expression. This further, through tumor angiogenesis disruption acts as anticancer agent. This decreases the total blood vessels numbers and causes a dramatic change along with the perfused vessels which indicates the improved efficacy against the tumor and vascular effects [13]. Efforts have been made to modify the influenza virus genome using recombinant DNA technology for development of vaccines. The modifications are based on engineering of vectors to expression of foreign genes. In practical, the NS gene of the influenza virus was replaced with foreign gene, commonly chloramphenicol acetyltransferase gene. Thereafter, the RNA previously recombined is expressed and packaged into virus particles after transfection with purified influenza A virus in the presence of helper virus. It has been clarified that 5′ terminal and the 3′ terminal bases are sufficient from influenza A virus RNA to produce signals for RNA replication, RNA transcription, and RNA packaging into influenza virus [15].

The abovementioned new production systems enhance pipelines for development of various vaccines and drugs and so forth. Production of high quality proteins depends on physiology of a cell and the conditions provided to it. The expression of proteins becomes retarded if a cell goes under stressful conditions, which may also favor the production in some cases. Thus, further improvements are required for the better and safe production at genetic and metabolic levels. Microorganisms are considered the most convenient hosts to produce molecular medicines. These cells allow the incorporation of foreign genes with less resistant barriers and expression is easily controlled. Compared to plant and mammalian cells to be taken as hosts, microbial systems provide less complicated machinery which ultimately enhances the performance and quality of proteins production. The use of common microbial species, including bacteria and yeasts, is promising but the less common strains have also been observed promising as being cellular factories to produce recombinant molecular drugs. The increasing demands of drugs and the needs of quality can be fulfilled with better results if these cellular factories of microorganisms get incorporated into productive processes of pharmaceuticals (Table 1) [41, 45, 46].

## 4. Applications of Recombinant DNA Technology

### 4.1. Food and Agriculture

Recombinant DNA technology has major uses which made the manufacturing of novel enzymes possible which are suitable in conditions for specified food-processing. Several important enzymes including lipases and amylases are available for the specific productions because of their particular roles and applications in food industries. Microbial strains production is another huge achievement that became possible with the help of recombinant DNA technology. A number of microbial strains have been developed which produce enzyme through specific engineering for production of proteases. Certain strains of fungi have been modified so that their ability of producing toxic materials could be reduced [47]. Lysozymes are the effective agents to get rid of bacteria in food industries. They prevent the colonization of microbial organisms. It is suitable agent for food items including fruits, vegetables, cheese, and meat to be stored as it increases their shelf life. The inhibition of food spoiling microorganisms can be carried out through immobilized lysozyme in polyvinyl alcohol films and cellulose. Lysozyme impregnation of fish skin gelatin gels increase the shelf life of food products and inhibit different food spoiling bacterial growth [48–50]. Exopolysaccharides of* Staphylococcus* and* E. coli* can be hydrolyzed with the use of DspB which is engineered from T7. This ability of DspB causes a declination in the bacterial population [50]. Biofilms related to food industries can be removed by the combining activity of serine proteases and amylases [51].* S. aureus*,* Salmonella infantis*,* Clostridium perfringens*,* B. cereus*,* Campylobacter jejuni*,* L. monocytogenes*,* Yersinia enterocolitica*, and some other food spoiling microorganisms can be inhibited by glucose oxidase. It is also considered one of the most important enzymes in food industry to kill wide range of foodborne pathogens [50].

Derivation of recombinant proteins being used as pharmaceuticals came into practice from first plant recently and many others are through to be used for more production of similar medically important proteins [52].

Wide range of recombinant proteins have been expressed in different plant species to be used as enzymes in industries, some majorly used proteins in research are proteins present in milk which play a role in nutrition, and new polymeric proteins are being used in industries and medical field [52]. With the invention of HBV vaccine production in plants, the oral vaccination concept with edible plants has gained popularity. Plants have been used to produce several therapeutic protein products, such as casein and lysozyme for improving health of child and polymers of protein for tissue replacement and surgery. Furthermore, tobacco plants can be engineered genetically to produce human collagen. High yielding molecular proteins is one of the major tasks under consideration in field of recombinant DNA technology [52]. Traditional breeding and quantitative trade locus (QTL) analysis assisted in the identification of a rice variety with protein kinase known as PSTOL1 (*phosphorus starvation tolerance1*) help in enhancing root growth in early stages and tolerates phosphorus deficiency [53]. Overexpression of this enzyme enables root to uptake nutrients in sufficient amount in phosphorus deficient soil which ultimately enhances the grain yield [54]. Chloroplast genome sequences are important in plant evolution and phylogeny.* Rpl22* is considered to be transferred from chloroplast into nuclear genome. This gene contains a peptide which plays role in delivery of protein from cytosol to chloroplast. A number of important genes deleted from chloroplast have been observed to be transferred into nucleus, except ycf1 and ycf2, in order to avoid disruptions in photosynthesis and other necessary processes. Trans-genesis into chloroplast is considered stable as the nuclear transgenic plants face the problems of lower expression and transgene escape via pollen. Almost ten thousand copies of transgenes have been incorporated into the genome of chloroplast [55–57]. Transgene expression is dependent on heterologous regulatory sequences but independent of cellular control. T7gene10 engineering against salt stress has been found successful but with lower expression rate into nongreen tissues. *γ*-tmt gene insertion into chloroplast genome results in multiple layer formation of the inner chloroplast envelope. Lycopene *β*-cyclase genes introduction into the plastid genome of tomato enhances the lycopene conversion into provitamin A [57, 58].

Organ or tissue specific genes identification can be carried out through gene expression profiles. cDNAs with full lengths are the main resources for expression profiling of genes. 44 K Agilent Oligonucleotide microarray is used for field grown rice transcriptome analysis. Gene expression fluctuation and transcriptome dynamics can be predicted by transcriptomic data and meteorological information. These processes and predictions are helpful to improve crop production and resistance to either environmental or microbial stresses. Resistance to fungal and bacterial infections can be enhanced by WRKY45 gene in rice which is induced by plant activator benzothiadiazole that activates innate immune system of plant. The larger grain size can be achieved by inserting qSW5 gene. qSH1 causes the loss of seed shattering by preventing the abscission layer formation. Kala4 gene is responsible for the black color of rice which makes the rice resistant to attacking pathogens [59, 60]. Genetic modification is needed in facilitating gene by gene introduction of well-known characters. It allows access to extended range of genes from an organism. Potato, beans, eggplant, sugar beet, squash, and many other plants are being developed with desirable characters, for example, tolerance of the herbicide glyphosate, resistance to insects, drought resistance, disease and salt tolerance. Nitrogen utilization, ripening, and nutritional versatility like characters have also been enhanced [61].

### 4.2. Health and Diseases

Recombinant DNA technology has wide spectrum of applications in treating diseases and improving health conditions. The following sections describe the important breakthroughs of recombinant DNA technology for the improvement of human health:

#### 4.2.1. Gene Therapy

Gene therapy is an advanced technique with therapeutic potential in health services. The first successful report in field of gene therapy to treat a genetic disease provided a more secure direction toward curing the deadliest genetic diseases [62, 63]. This strategy shows good response in providing treatment for adenosine deaminase-deficiency (ADA-SCID), which is a primary immunodeficiency. At the beginning of this technology, several challenges including maintenance of patients on PEGylated ADA (PEG-ADA) during gene therapy and the targeting of gene transfer to T-lymphocytes were the reasons for unsuccessful results [64, 65]. However, later on successful results were obtained by targeting haematopoietic stem cells (HSCs) by using an improved gene transfer protocol and a myeloablative conditioning regime [66].

Adrenoleukodystrophy (X-ALD) and X-linked disorder are is possible through the expression of specific genes transferred by lentiviral vector, based on HIV-1 [67]. X-ALD protein expression indicates that gene-correction of true HSCs was achieved successfully. The use of lentiviral vector was made successful for the first time to treat genetic human disease [68]. Metastatic melanoma was treated through immunotherapy by enhancing the specific proteins expression during 2006. This success in the field of health sciences opened up new doors to extend the research to treat serious death causing diseases through immunotherapy [69]. Highly sustained levels of cells that were engineered for tumor recognition in blood using a retrovirus encoding a T-cell receptor in two patients up to 1 year after infusion resulted in regression of metastatic melanoma lesions. This strategy was later used to treat patients with metastatic synovial cell carcinoma [70]. Autologous T-cells were genetically modified to express a Chimeric Antigen Receptors (CAR) with specificity for the B-cell antigen CD19 for the treatment of chronic lymphocytic leukemia. Genetically modified cells undergo selective expansion for diseases such as SCID-X1 and ADA-SCID as a consequence of in vivo selection conferred by the disease pathophysiology despite the correction of only a modest number of progenitors. Combination of gene and drug therapy's potential has recently been highlighted in a trial seeking to confer chemoprotection on human HSCs during chemotherapy with alkylating agents for glioblastoma [71].

Gene transfer to a small number of cells at anatomically discrete sites is a targeted strategy that has the potential to confer therapeutic benefit. It showed impressive results for incurable autosomal recessive dystrophies such as congenital blindness and Leber congenital amaurosis (LCA). Swiss–German phase I/II gene therapy clinical trial aimed to treat chronic granulomatous disease in April 2006 that came up with success [72]. Mobilized CD34+ cells isolated from peripheral blood were retrovirally transduced and infused into the patient where two-thirds of the patients showed clear benefit from this treatment. After the treatment silencing of the transgene as a result of methylation of the viral promoter caused the severity of infection that leaded to the death of patient [73].

Many different cancers including lung, gynecological, skin, urological, neurological, and gastrointestinal tumors, as well as hematological malignancies and pediatric tumors, have been targeted through gene therapy. Inserting tumor suppressor genes to immunotherapy, oncolytic virotherapy and gene directed enzyme prodrug therapy are different strategies that have been used to treat different types of cancers. The p53, a commonly transferred tumor suppressor gene, is a key player in cancer treating efforts. In some of the strategies, p53 gene transfer is combined with chemotherapy or radiotherapy. The most important strategies that have been employed until now are vaccination with tumor cells engineered to express immunostimulatory molecules, vaccination with recombinant viral vectors encoding tumor antigens and vaccination with host cells engineered to express tumor antigens [19]. New fiber chimeric oncolytic adenoviruses vectors (Ad5/35-EGFP) offer an affective new anticancer agent for the better cure of hepatocellular carcinoma. A demonstration of these vectors through proper assaying was significant for transduction improvement and more progeny of the virus were produced in HCC. A higher level of transgenic expression was mediated and an enhanced antitumor effect was observed on in vitro HCC cells while keeping the normal cells protected against cytotoxicity. Tumor growth was also inhibited by utilizing this technology [74]. Cancer gene therapy has become more advanced and its efficacy has been improved in recent years [75].

Treatment of cardiovascular diseases by gene therapy is an important strategy in health care science. In cardiovascular field, gene therapy will provide a new avenue for therapeutic angiogenesis, myocardial protection, regeneration and repair, prevention of restenosis following angioplasty, prevention of bypass graft failure, and risk-factor management. Mutation in gene encoding WASP, a protein regulating the cytoskeleton, causes Wiskott-Aldrich Syndrome (inherited immunodeficiency). Its treatment requires stem cells transplantation; in case matched donors are unavailable the treatment is carried out through infusion of autologous HSPCs modified ex vivo by gene therapy [76]. Metastatic cancer can be regressed through immunotherapy based on the adoptive transfer of gene-engineered T-cells. Accurate targeting of antigens expressed by tumors and the associated vasculature and the successful use of gene engineering to retarget T-cells before their transfer into the patient are mainly focused on in this therapy [77]. Cancer cells often make themselves almost “invisible” to the immune system and its microenvironment suppresses T-cells survival and migration but genetic engineering of T-cells is the solution to these challenges. T-cells in cancer patients can be modified by recombining the genes responsible for cancer-specific antigens recognition, resistance to immunosuppression, and extending survival and facilitating migration to tumors [78]. Fusion between the genes echinoderm microtubule-associated protein like 4 (*EML4*) and anaplastic lymphoma kinase (*ALK*) is generated by an inversion on the short arm of chromosome confers sensitivity to ALK inhibitors. Vial-mediated delivery of the CRISPR/Cas9 system to somatic cells of adult animals induces specific chromosomal rearrangements [79].

Wnt signaling is one of the key oncogenic pathways in multiple cancers. Targeting the Wnt pathway in cancer is an attractive therapeutic approach, where LGK974 potently inhibits Wnt signaling, has strong efficacy in rodent tumor models, and is well-tolerated. Head and neck cancer cell lines with loss-of-function mutations in the Notch signaling pathway have a high response rate to LGK974 [80]. Codon-optimized gene, on the basis of coding sequence of the influenza virus hemagglutinin gene, was synthesized and cloned into a recombinant modified vaccinia virus Ankara (MVA). Immunization with MVA-H7-Sh2 viral vector in ferrets proved to be immunogenic as unprotected animals that were mock vaccinated developed interstitial pneumonia and loss of appetite and weight but vaccination with MVA-H7-Sh2 protected the animals from severe disease [81]. Viral gene therapy is one of the leading and important therapies for head and neck cancer. Tumor-associated genes are targeted by viruses, and p53 gene function was targeted through such therapy at first. Cancer cells can be destroyed by oncolytic viruses through viral replication and by arming with therapeutic transgenes [82].

High density lipoprotein gene* ABCA1* mutation in cells can make the cells be differentiated into macrophages. Gene knockouts in embryonic stem cells enhance the capability of cells to be differentiated into macrophages and specifically target the desired pathogens. The allele replacements in this case will assist in studying protein coding changes and regulatory variants involved in alteration of mRNA transcription and stability in macrophages [83].

#### 4.2.2. Production of Antibodies and Their Derivatives

Plant systems have been recently used for the expression and development of different antibodies and their derivatives. Most importantly, out of many antibodies and antibody derivatives, seven have reached to the satisfactory stages of requirements. Transgenic tobacco plants can be used for the production of chimeric secretory IgA/G known as CaroRx, CaroRx. Oral pathogen responsible for decay of a tooth known as Streptococcus mutants, can be recognized by this antibody. A monoclonal antibody called T84.66 can affectively function to recognize antigen carcinoembryonic, which is still considered an affectively characterized marker in cancers of epithelia [84, 85]. A full-length humanized IgG1 known as anti-HSV and anti-RSV, which can function as the recognizing agent for herpes simplex virus (HSV)-2-glycoprotein B, has been expressed in transgenic soybean and Chinese Hamster Ovary (CHO) cells. Antibodies from both sources have been shown to prevent vaginal HSV-2 transmission in mice after applying topically; if worked similarly in humans it would be considered as inexpensive and affective prevention against diseases transmitted through sexual interactions [86–88].* 38C13* is scFv antibody based on the idiotype of malignant B lymphocytes in the well-characterized mouse lymphoma cell line 38C13. Administration of the antibody to mice resulted in the production of anti-idiotype antibodies that are able to recognize 38C13 cells, which help to protect the mice against with injected lymphoma cells, is a lethal challenge [89, 90]. Unique markers recognizing enzymes could be produced through this system, most affectively the surface markers of a malignant B-cells to work as an effective therapy for non-Hodgkin lymphoma like diseases in human [61]. A monoclonal antibody known as PIPP is specific for human chorionic gonadotropin recognition. The production of full-length monoclonal antibody and scFv and diabody derivatives was made possible in plants through transgenesis and agroinfiltration in tobacco transformed transiently [91]. Testosterone production by stimulated hCG can be inhibited by each of these antibodies in cells cultured by LEYDIG and uterine weight gain could be delayed in mice, through which hCG activity is checked. Diagnosis and therapy of tumors can be carried out with the help of antibodies [61].

#### 4.2.3. Investigation of the Drug Metabolism

Complex system of drug metabolizing enzymes involved in the drug metabolism is crucial to be investigated for the proper efficacy and effects of drugs. Recombinant DNA approaches have recently contributed its role through heterologous expression, where the enzyme's genetic information is expressed in vitro or in vivo, through the transfer of gene [92, 93].

#### 4.2.4. Development of Vaccines and Recombinant Hormones

Comparatively conventional vaccines have lower efficacy and specificity than recombinant vaccine. A fear free and painless technique to transfer adenovirus vectors encoding pathogen antigens is through nasal transfer which is also a rapid and protection sustaining method against mucosal pathogens. This acts as a drug vaccine where an anti-influenza state can be induced through a transgene expression in the airway [74].

In vitro production of human follicle-stimulating hormone (FSH) is now possible through recombinant DNA technology. FSH is considerably a complex heterodimeric protein and specified cell line from eukaryotes has been selected for its expression. Assisted reproduction treatment through stimulating follicular development is an achievement of recombinant DNA technology. A large number of patients are being treated through r-FSH. Most interestingly r-FSH and Luteinizing Hormone (LH) recombination was made successful to enhance the ovulation and pregnancy [94, 95].

#### 4.2.5. Chinese Medicines

As an important component of alternative medicine, traditional chines medicines play a crucial role in diagnostics and therapeutics. These medicines associated with theories which are congruent with gene therapy principle up to some extent. These drugs might be the sources of a carriage of therapeutic genes and as coadministrated drugs. Transgenic root system has valuable potential for additional genes introduction along with the Ri plasmid. It is mostly carried with modified genes in* A. rhizogenes* vector systems to enhance characteristics for specific use. The cultures became a valuable tool to study the biochemical properties and the gene expression profile of metabolic pathways. The intermediates and key enzymes involved in the biosynthesis of secondary metabolites can be elucidated by the turned cultures [96, 97].

#### 4.2.6. Medically Important Compounds in Berries

Improvement in nutritional values of strawberries has been carried through rolC gene. This gene increases the sugar content and antioxidant activity. Glycosylation of anthocyanins requires two enzymes glycosyl-transferase and transferase. Some nutrition related genes for different components in strawberry including proanthocyanidin, l-ascorbate, flavonoid, polyphenols, and flavonoid are important for improving the component of interest through genetic transformation. In case of raspberry, bHLH and FRUITE4 genes control the anthocyanin components whereas ERubLRSQ072H02 is related to flavonol. By specific transformation, these genes can enhance the production and improve the quality. All these mentioned compounds have medical values [98].

### 4.3. Environment

Genetic engineering has wide applications in solving the environmental issues. The release of genetically engineered microbes, for example,* Pseudomonas fluorescens* strain designated HK44, for bioremediation purposes in the field was first practiced by University of Tennessee and Oak Ridge National Laboratory by working in collaboration [99, 100]. The engineered strain contained naphthalene catabolic plasmid pUTK21 [101] and a transposon-based bioluminescence-producing* lux* gene fused within a promoter that resulted in improved naphthalene degradation and a coincident bioluminescent response [102]. HK44 serves as a reporter for naphthalene bioavailability and biodegradation whereas its bioluminescence signaling ability makes it able to be used as an online tool for in situ monitoring of bioremediation processes [102]. The production of bioluminescent signal is detectable using fiber optics and photon counting modules [101].

#### 4.3.1. Phytoremediation and Plant Resistance Development

Genetic engineering has been widely used for the detection and absorption of contaminants in drinking water and other samples. For example, At*PHR1* gene introduction into garden plants* Torenia*,* Petunia*, and* Verbena* changed their ability for Pi absorption. The At*PHR1* transgenic plants with enhanced Pi absorption ability can possibly facilitate effective phytoremediation in polluted aquatic environments [103]. A fragment of the At*PHR1* gene was inserted into binary vector pBinPLUS, which contains an enhanced cauliflower mosaic virus 35S promoter. This plasmid was named pSPB1898 and was used for transformation [104] in Petunia and Verbena using* Agrobacterium tumefaciens* [105]. At*PHR1* is effective in other plant species, such as* Torenia*,* Petunia*, and* Verbena* [103] but posttranscriptional modification of the endogenous At*PHR1* counterpart might be inhibited by overexpression of At*PHR1* [103].

Plant metabolism processes identify their importance to use for remediating the environmental pollutants. Some of the chemicals are not prone to be degraded or digested. TNT is only partially digested in which the nitrogen further reacts with oxygen to form toxic superoxide. To overcome this issue, the gene responsible for monodehydroascorbate reductase is knocked out which increases the plant tolerance against TNT. Fine-tuning enzymatic activity and knockout engineering together enhance the plant responses to toxic metals. Phytochelatin synthase, a heavy metal binding peptides synthesizing enzyme, revealed a way to enhance tolerance against heavy metals through enzymatic activity attenuation [106]. Recombinant DNA technology has proven to be effective in getting rid of arsenic particles that are considered as serious contaminants in soil. PvACR3, a key arsenite [As(III)] antiporter was expressed in* Arabidopsis* which showed enhanced tolerance to arsenic. Seeds of plants genetically engineered with* PvACR3* can germinate and grow in the presence of higher than normal quantity of arsenate [As(V)] which are generally lethal to wild-type seeds. Arsenic (As) is reduced by As reductase present in* A. thaliana*. Phytochelatins restrict the arsenic movement in root cells and phloem companion cells. OsNramp5 and OsHMA3 represent the transporters to uptake cadmium (Cd) and its retention [107]. In plants, brassino-steroid (BR) is involved in regulating physiological and developmental processes. Its activity is started with triggering phosphorylation or dephosphorylation cascade [108].

Recent biotechnological approaches for bioremediation include biosorption, phytostabilization, hyperaccumulation, dendroremediation, biostimulation, mycoremediation, cyanoremediation, and genoremediation, which majorly depend on enhancing or preventing specified genes activities. However, the challenges in adopting the successful technique cannot be ignored [109].

#### 4.3.2. Energy Applications

Several microorganisms, specifically cyanobacteria, mediate hydrogen production, which is environmental friendly energy source. The specific production is maintained by utilizing the required enzymes properly as these enzymes play a key role in the product formation. But advanced approaches like genetic engineering, alteration in nutrient and growth conditions, combined culture, metabolic engineering, and cell-free technology [110–112] have shown positive results to increase the hydrogen production in cyanobacteria and other biofuels [3, 4]. The commercialization of this energy source will keep the environment clean which is not possible by using conventional energy sources releasing CO_2_ and other hazardous chemicals [113]. Also cyanobacteria can be engineered to make them able to convert of CO_2_ into reduced fuel compounds. This will make the carbon energy sources harmless to environment. This approach has been successful for vast range of commodity chemicals, mostly energy carriers, such as short chain and medium chain alcohols [114].

The conductive biofilms of* Geobacter sulfurreducens* are potential sources in the field in renewable energy, bioremediation, and bioelectronics. Deletion of PilZ genes encoding proteins in* G. sulfurreducens* genome made the biofilm more active as compared to wild-type. CL-1ln is specified for the strain in which the gene GSU1240 was deleted. Biofilm production was enhanced along with the production of pili and exopolysaccharide. The electron acceptor CL-1 produced biofilms that were 6-fold more conductive than wild-type biofilms when they were grown with electrode. This high fold conductivity lowered the potential losses in microbial fuel cells, decreasing the charge transfer resistance at the biofilm-anode surface and lowering the formal potential. Potential energy was increased by lower losses [115].

## 5. Current Challenges and Future Prospects

The fact that microbial cells are mostly used in the production of recombinant pharmaceutical indicates that several obstacles come into their way restricting them from producing functional proteins efficiently but these are handled with alterations in the cellular systems. Common obstacles which must be dealt with are posttranslational modifications, cell stress responses activation, and instability of proteolytic activities, low solubility, and resistance in expressing new genes. Mutations occurring in humans at genetic levels cause deficiencies in proteins production, which can be altered/treated by incorporation of external genes to fill the gaps and reach the normal levels. The use of* Escherichia coli* in recombinant DNA technology acts as a biological framework that allows the producers to work in controlled ways to technically produce the required molecules through affordable processes [41, 116].

Recombinant DNA research shows great promise in further understanding of yeast biology by making possible the analysis and manipulation of yeast genes, not only in the test tube but also in yeast cells. Most importantly, it is now possible to return to yeast by transformation with DNA and cloning the genes using a variety of selectable marker systems developed for this purpose. These technological advancements have combined to make feasible truly molecular as well as classical genetic manipulation and analysis in yeast. The biological problems that have been most effectively addressed by recombinant DNA technology are ones that have the structure and organization of individual genes as their central issue [117, 118]. Recombinant DNA technology is recently passing thorough development which has brought tremendous changes in the research lines and opened directions for advanced and interesting ways of research for biosynthetic pathways though genetic manipulation.* Actinomycetes* are being used for pharmaceutical productions, for example, some useful compounds in health sciences and the manipulation of biosynthetic pathways for a novel drugs generation. These contribute to the production of a major part of biosynthetic compounds and thus have received immense considerations in recombinant drugs designing. Their compounds in clinical trials are more applicable as they have shown high level activity against various types of bacteria and other pathogenic microorganisms. These compounds have also shown antitumor activity and immunosuppressant activity [119].

Recombinant DNA tech as a tool of gene therapy is a source of prevention and cure against acquired genetic disorders collectively. DNA vaccines development is a new approach to provide immunity against several diseases. In this process, the DNA delivered contains genes that code for pathogenic proteins. Human gene therapy is mostly aimed to treat cancer in clinical trials. Research has focused mainly on high transfection efficacy related to gene delivery system designing. Transfection for cancer gene therapy with minimal toxicity, such as in case of brain cancer, breast cancer, lung cancer, and prostate cancer, is still under investigation. Also renal transplantation, Gaucher disease, hemophilia, Alport syndrome, renal fibrosis, and some other diseases are under consideration for gene therapy [120].

## 6. Conclusions

Recombinant DNA technology is an important development in science that has made the human life much easier. In recent years, it has advanced strategies for biomedical applications such as cancer treatment, genetic diseases, diabetes, and several plants disorders especially viral and fungal resistance. The role of recombinant DNA technology in making environment clean (phytoremediation and microbial remediation) and enhanced resistace of plants to different adverse acting factors (drought, pests, and salt) has been recognized widely. The improvements it brought not only in humans but also in plants and microorganisms are very significant. The challenges in improving the products at gene level sometimes face serious difficulties which are needed to be dealt for the betterment of the recombinant DNA technology future. In pharmaceuticals, especially, there are serious issues to produce good quality products as the change brought into a gene is not accepted by the body. Moreover, in case of increasing product it is not always positive because different factors may interfere to prevent it from being successful. Considering health issues, the recombinant technology is helping in treating several diseases which cannot be treated in normal conditions, although the immune responses hinder achieving good results.

Several difficulties are encountered by the genetic engineering strategies which needed to be overcome by more specific gene enhancement according to the organism's genome. The integration of incoming single-stranded DNA into the bacterial chromosome would be carried out by a RecA-dependent process. This requires sequence homology between both entities, the bacterial chromosome and incoming DNA. Stable maintenance and reconstitution of plasmid could be made easy. The introduction of genetic material from one source into the other is a disaster for safety and biodiversity. There are several concerns over development of genetically engineered plants and other products. For example, it is obvious that genetically engineered plants can cross-breed with wild plants, thus spreading their “engineered” genes into the environment, contaminating our biodiversity. Further, concerns exist that genetic engineering has dangerous health implications. Thus, further extensive research is required in this field to overcome such issues and resolve the concerns of common people.

## Figures and Tables

**Table 1:** Current DNA assembly methods for the synthesis of large DNA molecules. The table has been reproduced from Nature reviews 14: 781–793, with permission from Nature Publishing Group.

Method	Mechanism	Overhang (bp)	Scar (bp)	Comments	Examples of applications
BioBricks	Type IIP restriction endonuclease	8	8	Sequentially assembles small numbers of sequences	Construction of a functional gene expressing enhanced cyan fluorescent protein
BglBricks	Type IIP restriction endonuclease	6	6	Uses a highly efficient and commonly used restriction endonuclease, the recognition sequences of which are not blocked by the most common DNA methylases	Construction of constitutively active gene-expression devices and chimeric, multidomain protein fusions
Pairwise selection	Type IIS restriction endonuclease	65	4	Requires attachment tags at each end of fragments to act as promoters for antibiotic resistance markers; rapid, as a liquid culture system is used	Assembly of a 91 kb fragment from 1-2 kb fragments
GoldenGate	Type IIS restriction endonuclease	4	0	Allows large-scale assembly; ligations are done in parallel one-step assembly of 2-3 fragment	One-step assembly of 2-3 fragments
Overlapping PCR	Overlap	0	0	Uses overlapping primers for the PCR amplification of 1–3 kb-long fragments	Usually used for 1–3 kb-long fragments, for example, for gene cassette construction
CPEC	Overlap	20–75	0	Uses a single polymerase for the assembly of multiple inserts into any vector in a one-step reaction in vitro	One-step assembly of four 0.17–3.2 kb-long PCR fragments
Gateway	Overlap	20	0	Uses a specific recombinase for small-scale assembly	One-step assembly of three 0.8–2.3 kb-long fragments
USER	Overlap	Up to 708	0	Replaces a thymidine with a uracil in the PCR primers, which leaves 3′ overhangs for cloning after cleaving by a uracil exonuclease	One-step assembly of three 0.6–1.5 kb-long fragments
InFusion	Overlap	15	0	Uses an enzyme mix for parallel assembly through a “chew-back-and-anneal” method	One-step assembly of three 0.2–3.8 kb-long fragments
SLIC	Overlap	>30	0	(i) Uses a T4 DNA polymerase through a chew-back method in the absence of dNTPs (ii) Uses Recombinase A^*∗*^ to stabilize the annealed fragments and avoid in vitro ligation (iii) Allows the parallel assembly of several hundred base-long fragments	Generation of a ten-way assembly of 300–400 bp-long PCR fragments
Gibson	Overlap	40–400	0	Uses enzymatic “cocktails” to chew back and anneal for the parallel assembly of several kilobase-long fragments	Assembly of the 1.08 Mb *Mycoplasma mycoides* JCVI-syn1.0 genome
